# Clinical Symptoms and Types of Samples Are Critical Factors for the Molecular Diagnosis of Symptomatic COVID-19 Patients: A Systematic Literature Review

**DOI:** 10.1155/2021/5528786

**Published:** 2021-09-06

**Authors:** Milad Zandi, Abbas Farahani, Armin Zakeri, Sara Akhavan Rezayat, Ramin Mohammadi, Umashankar Das, Jonathan R. Dimmock, Shervin Afzali, Mohammadvala Ashtar Nakhaei, Alireza Doroudi, Yousef Erfani, Saber Soltani

**Affiliations:** ^1^Department of Virology, School of Public Health, Tehran University of Medical Sciences, Tehran, Iran; ^2^Students' Scientific Research Center, Tehran University of Medical Sciences, Tehran, Iran; ^3^Research Center for Clinical Virology, Tehran University of Medical Sciences, Tehran, Iran; ^4^Infectious and Tropical Diseases Research Center, Hormozgan Health Institute, Hormozgan University of Medical Sciences, Bandar Abbas, Iran; ^5^Department of Hematology, Faculty of Medical Sciences, Tarbiat Modares University, Tehran, Iran; ^6^Department of Health Economics and Management, School of Public Health, Tehran University of Medical Sciences, Tehran, Iran; ^7^Drug Discovery and Development Research Group, College of Pharmacy and Nutrition, University of Saskatchewan, Saskatoon, Canada; ^8^Department of Cellular and Molecular Biology, Faculty of Life Sciences and Biotechnology, Shahid Beheshti University G.C., Tehran, Iran; ^9^Department of Medical Laboratory Sciences, School of Allied Medical Sciences, Tehran University Medical Sciences, Tehran, Iran

## Abstract

**Background:**

Currently, a novel coronavirus found in 2019 known as SARS-CoV-2 is the etiological agent of the COVID-19 pandemic. Various parameters including clinical manifestations and molecular evaluation can affect the accuracy of diagnosis. This review aims to discuss the various clinical symptoms and molecular evaluation results in COVID-19 patients, to point out the importance of onset symptoms, type, and timing of the sampling, besides the methods that are used for detection of SARS-CoV-2.

**Methods:**

A systematic literature review of current articles in the Web of Science, PubMed, Scopus, and EMBASE was conducted according to the PRISMA guideline.

**Results:**

Of the 12946 patients evaluated in this investigation, 7643 were confirmed to be COVID-19 positive by molecular techniques, particularly the RT-PCR/qPCR combined technique (qRT-PCR). In most of the studies, all of the enrolled cases had 100% positive results for molecular evaluation. Among the COVID-19 patients who were identified as such by positive PCR results, most of them showed fever or cough as the primary clinical signs. Less common symptoms observed in clinically confirmed cases were hemoptysis, bloody sputum, mental disorders, and nasal congestion. The most common clinical samples for PCR-confirmed COVID-19 patients were obtained from throat, oropharyngeal, and nasopharyngeal swabs, while tears and conjunctival secretions seem to be the least common clinical samples for COVID-19 diagnosis among studies. Also, different conserved SARS-CoV-2 gene sequences could be targeted for qRT-PCR detection. The suggested molecular assay being used by most laboratories for the detection of SARS-CoV-2 is qRT-PCR.

**Conclusion:**

There is a worldwide concern on the COVID-19 pandemic and a lack of well-managed global control. Hence, it is crucial to update the molecular diagnostics protocols for handling the situation. This is possible by understanding the available advances in assays for the detection of the SARS-CoV-2 infection. Good sampling procedure and using samples with enough viral loads, also considering the onset symptoms, may reduce the qRT-PCR false-negative results in symptomatic COVID-19 patients. Selection of the most efficient primer-probe for target genes and samples containing enough viral loads to search for the existence of SARS-CoV-2 helps detecting the virus on time using qRT-PCR.

## 1. Introduction

Coronaviruses are enveloped, nonsegmented, positive-sense RNA viruses. They belong to Coronaviridae and Coronavirinae, which are divided into four genera, namely, alpha, beta, gamma, and delta coronaviruses [1]. The genus *Betacoronavirus* comprises five subgenera including *Embecovirus*, *Sarbecovirus*, *Merbecovirus*, *Nobecovirus*, and *Hibecovirus* [2]. On March 2, 2020, a coronaviridae study group of the international committee on taxonomy of viruses (ICTV) declared their decision regarding the change of the name of the novel coronavirus (formerly known as 2019-nCoV) to severe acute respiratory syndrome corona virus 2 (SARS-CoV-2) which is accountable for the COVID-19 pandemic [3]. Coronaviruses did not require serious considerations before the SARS-CoV outbreak in 2002 [4], since they mainly cause common cold and diarrhea [5]. Subsequent to the SARS-CoV outbreak in 2002, there was another coronavirus outbreak in 2012, i.e., MERS-CoV [6]. MERS-CoV was first isolated from a 60-year-old man from Saudi Arabia (previously known as HCoV-EMC) [7] and including the last reported case on February 17, 2020, when the World Health Organization (WHO) declared that there were 2521 laboratory-confirmed cases with 866 deaths (34.4% case fatality rate) [8]. Different diseases which cause pneumonia often have the same symptoms, which makes it hard to determine the exact cause of the disease in order to start medication, and the disease caused by SARS-CoV-2 is not an exception. The main symptoms for COVID-19 are reported to be fever and fatigue, followed by sore throat and dyspnea and even, in some cases, neurological symptoms [9–11]. As the knowledge about coronaviruses causing diseases in humans was insufficient in 2002, the early diagnostic tests for SARS-CoV were not wholly reliable. The advances toward a more sensitive molecular method have solved the problem. Not only is the procedure itself of importance but also the types of the specimen prepared for the tests were. For instance, nasal wash specimens, throat swabs, and sputum were better at the onset of the infection [12]. The criterion for considering a patient as a SARS-CoV-infected case was the body temperature of more than 37.5 C and having a nonproductive cough or dyspnea. The mainstay of confirmed diagnoses was the reverse transcriptase-polymerase chain reaction (RT-PCR) along with positive acute or serology tests [13–15]. RT-PCR has also been used for confirmation of MERS-CoV cases [16]. The WHO announced that regular confirmation of COVID-19 cases is possible by analyzing RT-R-PCR results of N, E, S, and RdRp genes (unique genes) [17]. The biosensor techniques can help COVID-19 diagnosis [18]. According to the previous infections by SARS-CoV and MERS-CoV, it is of importance to detect the SARS-CoV-2-infected individuals early to hinder the further spread of the virus, and it could be managed by utilizing the most beneficial detecting method along with the right sample. Also, using the most sufficient gene for RT-real-time PCR (as the molecular method) and considering the onset symptoms is of great importance in the process of detecting the infected patients. This study aims to point out the importance of clinical manifestations, detection methods of SARS-CoV2, and also timing and the types of samples in better detection of infected individuals with the aim of restricting the spread of the virus.

## 2. Materials and Methods

### 2.1. Data Sources and Search Strategies

**Figure 1:**
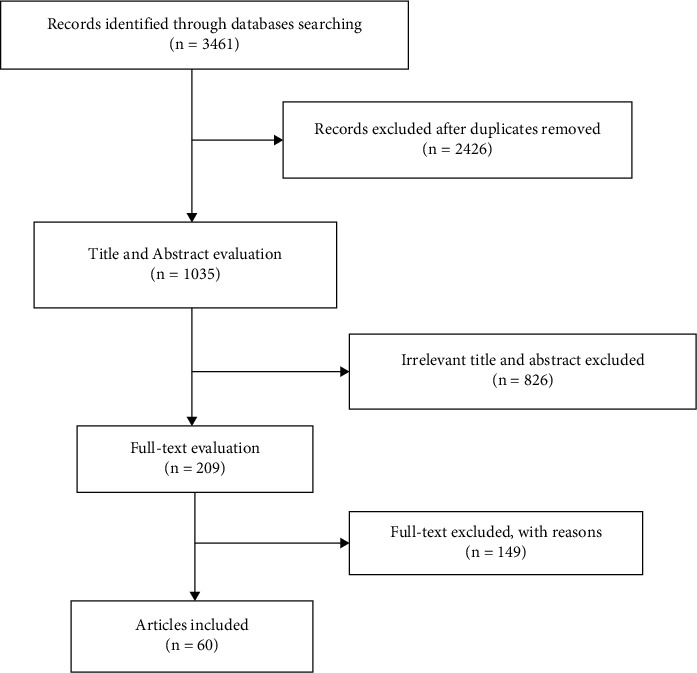
PRISMA 2009 flow diagram and summary of the literature search and study selection.

We conducted a systematic literature review using Web of Science, Medline/PubMed, Scopus, and EMBASE according to the PRISMA guideline [19]. The survey was concluded from 1 January 2020 until the end of April 2020. Three different researchers independently reviewed the search results. According to medical subject headings (MeSH), the following search keywords were used: “Novel coronavirus 2019,” “2019 nCoV,” “COVID-19,” Coronavirus disease 2019,” “Wuhan coronavirus,” “SARS-CoV-2,” “severe acute respiratory syndrome coronavirus 2,” “PCR,” “Real-time PCR,” “Symptoms,” and “Clinical manifestation.” The search results and study selection are shown in Figure 1.

### 2.2. Study Selection

Duplicate records were removed, and the results of the search were screened according to the titles and abstracts. In the next step, the English language full texts of articles were examined for inclusion and exclusion criteria (Figure 1). Articles that reported duplicate data from the same patients were excluded. The following articles with molecular data as well as clinical signs were selected in this study.

### 2.3. Data Collection Process and Data Items

The data extraction tables included information on the names of the authors, the number of patients, clinical symptoms (e.g., fever, cough, and fatigue), and molecular evaluation (e.g., sample type, PCR result, and target gene) which was extracted independently by three researchers. A fourth researcher screened the final data extraction list to prevent bias. Conflicts were decided by a fifth expert investigator.

### 2.4. Inclusion Criteria

We evaluated and included the published peer-review articles, which reported related clinical symptoms and the result of real-time reverse transcriptase-polymerase chain reaction (RRT-PCR) data for COVID-19 patients. For assessing clinical and molecular characteristics, eligible study designs such as series studies, case-control studies, and cohort studies were included. Article language limit was set on English, but we evaluated the non-English articles with essential data from their English abstracts.

### 2.5. Exclusion Criteria

Some studies were excluded due to the lack of relevant data or not meeting eligibility criteria. Articles which only assessed COVID-19 patients without molecular data were excluded. Literature reviews were screened for relevant citations, and letters to the editor, opinion articles, and case reports were excluded.

### 2.6. Quality Assessment

The quality of the chosen articles was evaluated using a checklist that covered the type of study, patients' number, sample type, real-time PCR result, target gene, data collection tools, and results analysis. Each article receives a score for the characteristic of a satisfactory methodology.

## 3. Results

In this section, we tried to mention the percentage of PCR-confirmed COVID-19 patients among the whole patients in the different enrolled studies and also tried to mention the results of molecular evaluation for detecting SARS-CoV-2 from the enrolled articles. We also mentioned the distribution of the different clinical manifestations among the confirmed patients. Then, we mentioned the results of our analysis about the different types of the samples and also the targeted genes that are used for the molecular detection of SARS-CoV-2 by RT-real-time PCR.

### 3.1. General and Demographic Data of Studies and Patients

The initial literature searching on databases collected 3461 articles. After checking the titles and abstracts and removing duplicate ones, 3252 citations were excluded. Finally, 60 articles met our inclusion criteria and were eligible for remaining in our systemic review. These articles were incorporated into the Endnote library for further investigations. The summary of the study selection strategy information is presented in Figure 1. A total of 60 articles were published in 2020. In 58 citations, both abstracts and full texts were in English while two studies had English abstracts and Chinese full texts.

Patients who suffered from COVID-19 show various types of symptoms. According to our screening of 60 selected articles, the highest prevalence rate was fever (100%). The majority of the citations were conducted in China. The total number of patients involved in this investigation was 12946 (men and women) while 7643 of them were confirmed to be COVID-19 positive by molecular techniques, particularly the reverse transcription-polymerase chain reaction (RT-PCR) (Table 1).

### 3.2. Clinical Symptoms and Clinical Features of Patients

Patients who suffered from COVID-19 show various types of symptoms. According to our screening of 60 selected articles, the highest prevalence rate was of fever (100%) [77], while the lowest was 25%, and the prevalence rate of cough was 91% among 60 evaluated citations. The proportion of patients who had fatigue and myalgia ranged from 5.9% to 75% and 3.33% to 70%, respectively. Chest pain/tightness was reported in 11 studies. Abdominal pain was documented in 6 studies, and mental disorders and confusion were observed in 2 citations only with 16.25% and 9% prevalence rate, respectively [37, 79]. In 7 studies, dizziness was a less common symptom in patients. Two studies reported nasal congestion by different rates of 61.5% and 6.9% [31, 78]. Other clinical symptoms such as shortness of breath (dyspnea), rhinorrhea, expectoration, chills and sore throat, nausea or vomiting, diarrhea, headache, and anorexia were less frequent in these studies. In 9 investigations, clinical manifestations were not documented.

### 3.3. Molecular Evaluation (RT-Real-Time PCR) Results

The RT-PCR results of 60 articles have been collected carefully. Several cohort and retrospective studies relied on RT-PCR results of other articles, and some conducted molecular evaluation by themselves. Among 12946 patients incorporated into our analysis, with 6 to 4880 patients per study, the COVID-19 RT-R-PCR assays of 7643 were positive. In most citations, all of the patients had 100% positive results for molecular evaluation. In the other cases, a fluctuation in their PCR positivity rate reports was noted. Three studies had positive RT-PCR assays below 10% [53, 54, 74]. While 40 articles recorded positive results over a 90% rate, 17 citations declared their positivity proportion between 10 and 90%.

### 3.4. RT-R-PCR Results and Clinical Symptoms

In almost all the citations which showed positivity in their PCR results, fever and cough were their main clinical manifestations while hemoptysis and blood in the sputum were the least common ones. Most of the positive cases experienced dyspnea, myalgia, nausea or vomiting, diarrhea, expectoration, chest pain, and headache. In one study, 4 out of 6 patients showed COVID-19 nucleic acid in their clinical samples; fever was their common symptom, while their other specific manifestations were cough, pharyngalgia, myalgia, and diarrhea [27]. The positivity rate of PCR was 96%, and the patients showed poor appetite besides fever, cough, and fatigue [47]. Furthermore, in two other studies with 100% positivity in the presence of COVID-19 in the samples, poor appetite or loss of appetite were common symptoms among patients [55, 61]. Based on two studies with 100% and one study with 61% positive PCR results, anorexia was a common manifestation [32, 38, 73]. According to Wu et al., mental disorder was diagnosed among 13 out of 80 COVID-19 positive patients besides fever, cough, headache, muscle ache, diarrhea, and chest pain [79]. Furthermore, an article by Chen et al. revealed 100% positivity in the PCR results while confusion was observed with a 9% prevalence rate besides other symptoms [37].

### 3.5. RT-R-PCR Results and Sample Types

Various samples were obtained from different sites of patients' bodies for COVID-19 diagnosis. Regarding the PCR results with 100% positivity for the presence of COVID-19 nucleic acid, the most common clinical samples were throat, oropharyngeal, and nasopharyngeal swabs. In three studies, all of the patients detected with COVID-19 [20, 23, 66] and four other studies with different prevalences of COVID-19-infected patients, urine samples were employed in their molecular assays and the positivity results had much fluctuation in comparison to each other. One citation with a 100% PCR positivity rate [20] and 4 citations with different rates of positivity used stool samples to detect COVID-19 of their cases. In two studies with 77% and 91.7% positive PCR results, saliva was used for their molecular assay [22, 72]. Xia et al. worked on tears and conjunctival secretions of 30 COVID-19 positive patients, and their PCR results found only one patient had positive results in conjunctival swab samples [53]. In two citations, rectal swabs were collected for COVID-19 diagnosis and showed 100% and 77% PCR positive results [66, 72]. Five citations used bronchoalveolar lavage samples for molecular evaluation and the rate of COVID-19 prevalence in three of them was 100% [21, 42, 44], and two other results reported 69% and 44% [26, 69]. The study conducted by Chung et al. and Bergheim et al. on the endotracheal aspirates of patients were found to be COVID-19 positive by PCR assay.

Various studies that employed sputum samples in their molecular investigation revealed that most of them showed 100% positivity in the PCR results and only three studies had 69%, 30.5%, and 7% positive prevalence rates.

### 3.6. RT-R-PCR Results and Target Gene

Different conserved SARS-CoV-2 gene sequences could be targeted for RT-PCR detection. In our systematic review analysis, 18 out of 60 included studies using several COVID-19 genes for diagnosing the presence of the virus nucleic acid in the samples of patients while more than half of the citations (*n* = 42) did not mention target genes for their PCR analysis. Nine citations used ORF1b [26, 36], and one study used ORF4 [69]. PCR target genes have different PCR positivity rates ranging from 38.5% to 100% per study. Based on 12 studies using the nucleocapsid (N) sequence as the PCR-specific gene, 8 out of 12 studies found COVID-19 nucleic acid in all of their patients' samples. Four citations used spike (S) gene sequence as the PCR target. This gene was present in all of the samples of the patients in 2 studies [40, 66], and in two other studies, they found 44.6% and 91.7% PCR positivity rates [22, 69]. Two studies revealed that envelope gene sequence was their target; all of the patients were infected with COVID-19 and showed positive results in the molecular assays [21, 34]. Also, the RNA-dependent RNA-polymerase (RdRp) gene was the PCR specific gene with positive results [22, 66].

## 4. Discussion

### 4.1. A Lookback on Previous Members of Coronaviridae

SARS-CoV, MERS-CoV, and SARS-CoV-2 have proven to be threats against humans since they took so many lives during the past two decades. Other coronaviruses, such as HCoV-229E, HCoV0HKU1, HCoV-NL63, and HCoV-OC43, are responsible for mild-to-moderate lower respiratory tract infections, including bronchiolitis and pneumonia [80]. HCoV-NL63 mostly causes illness in children and the elderly which has been shown to be associated with less than 10% of respiratory tract infections in children [81]. These viruses' fatality may not be like that of SARS-CoV and MERS-CoV and SARS-CoV-2. Nevertheless, it is crucial to detect the causative agent. To be able to control pathogens in order not to spread the viruses and risk any more lives, several diagnostic techniques were developed to take the situation under control. Before undergoing diagnostic testing, it was suggested that onset symptoms (i.e., fever, fatigue, cough, and dyspnea) and clinical features should be considered which are the most common along with pneumonia [82, 83]. In MERS-CoV cases, it was shown that, among three groups of patients, the ones exposed to the virus without any signs of pneumonia (group A) and patients with signs of pneumonia and respiratory failure (groups B and C, respectively), the ratio of patients with signs of lymphopenia was higher in group C compared to B and A (87.5%, 50.0%, and 9.1% for groups C, B, and A, respectively [84].

According to the WHO report, a suspected SARS case was for the first time described in 2003 with pneumonia which was supported by chest X-ray or a positive result by one or more assays (e.g., PCF, EKISA, and IF (A)) or autopsy results should be in accord with RDS pathology [85]. Despite a few amino acid differences in a few residues, including amino acids in the 8b and 3c proteins, the receptor-binding domain (RBD) of SARS-CoV-2 is somewhat similar to that of SARS-CoV. Also, the primary protease is highly conserved between these two. These similarities alleviated the problems of developing new diagnostic assays which were already available for SARS-CoV [86]. The blood parameters and symptoms monitored are not accurate to determine the disease status. The most common target genes for the SARS-CoV detection are nucleocapsid and polymerase (pol) genes [87]. The results should be normalized to the expression of internal control genes, including beta-actin and GADPH [88, 89].

### 4.2. Coronavirus Detection Methods and Different Types of Samples

Most citations included in this systematic review used the sequence of COVID-19 ORF genes in their investigations, and they accounted for the highest prevalence rate of COVID-19 infectivity in PCR assessments. Other genes of COVID-19 were employed in articles with various prevalence ranges of positivity in molecular tests. Despite the acceptance of RT-PCR as the main assay for confirmation of SARS-CoV infection, the sensitivity of RT-PCR is not as high as that of ELISA and IFA [90]. Ai et al. performed a study on 1014 cases with the purpose of indicating the correlation of chest CT and RT-PCR testing in COVID-19 patients. These scientists declared that, of 601 RT-PCR-positive COVID-19 patients, 580 were reported with positive CT scan results. In fact, Ai et al. suggested the sensitivity of 97% for CT scan based on positive RT-PCR results [41]. In another study on 51 patients, the comparison of RT-PCR and chest CT showed a sensitivity of 71–98%, respectively [45]. Above all, it seems pertinent to use a combination of molecular assays to achieve the integrity of the final results. Chest CT is also of great value for the detection of the onset of infection and observing its progress throughout the treatment [91]. After SARS-CoV's appearance in 2002, several assays were developed to detect the infection, i.e., fluorescent antibody detection, indirect ELISA, and RT-PCR. The indirect ELISA results have a 2% false-positive range, and the infection can only be confirmed when there is a transition from seronegative to seropositive [92]. Seroconversion has reported to happen 28 days after infection onset in 93% of patients diagnosed with SARS.

Retrospective detection of the SARS-CoV is useless because there is no time to undergo treatment [93]. Molecular detection assays are good tools for confirmation of coronavirus infection because of their sensitivity and specificity provided that we are aware of false results due to the existence of inconstant viral loads and inaccuracy while samples are being prepared [94]. Nevertheless, high-throughput detection test assays such as next-generation sequencing were used for origin confirmation of SARS-CoV-2 [95, 96]. There are several main reasons that justify the false-positive results of RT-PCR. First, at early stages after the disease onset, it is possible to get false-negative results from RT-R-PCR, and in that case, chest radiographs would be necessary to confirm the results [97]. Second, low viral load and laboratory errors might be playing a key role [54]. Specimen collection methods and choosing the right tissue for sample collection are also important in obtaining reliable results [28]. The suggested common specimens for the screening of SARS-CoV, MERS-CoV, and SARS-CoV-2 infections are frequently the same (i.e., nasopharyngeal and endotracheal aspirate, oropharyngeal saliva, and saliva) [72, 98–100]. The sputum sample needs to be mixed with 2 mL of phosphate-buffered saline (PBS). Subsequently, RNA should be extracted using available RNA extraction kits or nucleic acid isolation kit [101, 102]. In our analysis, the most common clinical samples among patients who were confirmed to be infected with COVID-19 is by PCR analysis were throat, oropharyngeal, and nasopharyngeal swabs while tears and conjunctival secretions seem to be the least common clinical sample for COVID -19 diagnosis among these 60 studies. The suggested molecular assay is being used by most laboratories for the detection of SARS-CoV-2 is qRT-PCR. Both qualitative and quantitative real-time RT-PCR are being applied as diagnostic tests for coronavirus infections [103, 104]. Quantitative real-time PCR (qRT-PCR) and high-throughput sequencing are two routinely used nucleic acid testing assays that are suitable for SARS-CoV-2. However, in comparison to high-throughput sequencing, qRT-PCR cost-effectiveness made it more common worldwide [105].

As SARS-CoV was the first coronavirus responsible for an outbreak in humans and because of the lack of insight into its genome, in most of the studies, the target genes used for PCR were segments of the POL1b coding region [106, 107]. Open reading frames (ORFs) 1a and 1b and the upstream of the envelope gene (upE) were used as targets for the detection of MERS-CoV [8, 100]. The recommended target genes for SARS-CoV-2 infection screening using RT-PCR mainly consist of orf1ab and nucleocapsid protein (NP) genes [77, 108]. Several genes were suggested for screening, including RDRP, E, and N genes [109]. Other minor coronaviruses (HCoV-229E, HCoV-HKU1, HCoV-NL63, and HCoV-OC43) have a low prevalence. Molecular detection of these viruses is based on the amplification of the membrane glycoprotein for 229E and OC43 and amplification of nucleocapsid protein for HKU1 and NL63 [80]. Notwithstanding the reported availability of COVID-19 PCR results, the lack of a clear correlation among qRT-PCR results, symptoms, sample types, and target genes is hindering the interpretation integrity of the PCR test results. That being said, recently, a new molecular detection method has been developed by Ishige et al. in which by the use of multiplex rRT-PCR, SARS-CoV-2 can be detected in clinical laboratories with high sensitivity. Not only does this method use two regions of the SARS-CoV-2 gene (E and N genes) for detection but also uses the human ABL1 gene as the inner control to evaluate the qualities of clinical specimens. In addition, these scientists mentioned that this method can diminish the usage of reagents and also it costs less [110]. Furthermore, despite the fact that detecting SARS-CoV-2 RNA in respiratory samples is considered to be the reference method, LO et al. proposed that utilizing both respiratory and fecal specimens can increase diagnostic sensitivity. As stool's viral load goes up in 2-3 weeks, meanwhile, SARS-CoV-2 RNA may not be detectable through respiratory samples [111]. In line with this study, Tang et al. performed RT-PCR on both stool and respiratory tract samples of a 10-year-old asymptomatic boy for detection of SARS-CoV-2 RNA and reported that, although RNA of SARS-CoV-2 was present in the stool sample, it was not detectable in the respiratory specimen [112].

### 4.3. Clinical Manifestations of COVID-19 Cases and Their Correlation with PCR Test Results

In this research letter, we attempted to gather the available data on PCR test results on COVID-19-suspected cases and summarize the significant trends. It is noteworthy to mention that, in 37 out of 60 articles, it showed positivity in all cases for the PCR test results. Considering the positive PCR results of suspected cases, the most prevalent observed symptoms include fever followed by cough which suggests that, during the time of the pandemic, patients who are showing these manifestations should be closely monitored until the real cause of the disease is determined [20, 21, 25]. Less common symptoms observed in clinically confirmed cases were hemoptysis, bloody sputum, mental disorders, and nasal congestion. These symptoms may be of low diagnostic value because of their frequency of presentation in COVID-19 cases. Nevertheless, it is of importance for its cause to undergo an investigation [52, 78, 79, 113]. Other frequent clinical manifestations including dyspnea, rhinorrhea, expectoration, chills, sore throat, nausea or vomiting, diarrhea, headache, and anorexia were also mentioned for SARS-CoV-2 [20, 21, 24, 32, 38, 39, 73, 110]. Regarding the clinical symptoms of COVID-19, infected patients' fever and cough were the most prevalent clinical manifestations in each study, while mental disorders, confusion, nasal congestion, hemoptysis, and bloody sputum were the least common clinical symptoms reported in these 60 citations. Other common clinical symptoms which had different prevalent rates include shortness of breath (dyspnea), rhinorrhea, expectoration, chills and sore throat, nausea or vomiting, diarrhea, headache, and anorexia. Nine studies did not mention any clinical manifestations of the patients, and so, this conclusion is based on the studies that mentioned their patient's clinical symptoms. Given the relationship between the main clinical symptoms and PCR results, these 9 studies were not considered since no clinical observations were mentioned.

The false-negative test results impede control over spreading the disease. As it is asserted that a major reason for that to happen is the availability of inadequate viral load in tissue samples, considering the time of symptom onset, the selection of the right type of specimen is necessary [54]. In a study, it was mentioned that some patients had negative NAAT results from pharyngeal swabs but tested positive for SARS-CoV-2 from bronchoalveolar lavage [27]. Among the clinical samples used for the PCR assay which subsequently resulted positive for COVID-19, nasopharyngeal, oropharyngeal, and throat swabs were the most common ones and conjunctival secretions and tears were infrequent [20, 42, 45, 53]. In 60 articles, 18 of them mentioned the target genes used for the PCR assay. In the majority of these articles, 11 employed ORF and nucleocapsid genes. These genes made a major contribution to the positive results retrieved from the PCR assays [66, 70, 73, 77]. Other genes, including S and E, were also used [40, 59]. Also, the RdRp/Hel gene was targeted. [66]. Rare symptoms, including hemoptysis and bloody sputum, were associated with PCR-positive results when respiratory tract samples were prepared for the test [52, 113]. Likewise, when fever and cough were the primary symptoms exerted on patients, the throat swab samples have a higher positivity rate in PCR test results [26, 31, 32].

### 4.4. Available Treatments

It seems that the only way the COVID-19 pandemic can be controlled is the creation of an effective vaccine and development of novel antiviral drugs. Due to the fact that creation of new drugs and definitive treatments are time consuming, various clinical trials are currently underway to reposition available drugs to control the rate of infection. In this regard, several studies have been undertaken to establish the effectiveness of chloroquine and hydroxychloroquine (HCQ) in the treatment of COVID-19 infection [114, 115]. Gautret et al. reported that administration of hydroxychloroquine, which can be reinforced by azithromycin, can decrease the viral load in 3 to 6 days after administration. The results suggest that this combination can reduce the viral load and consequently limit the transmission of the virus to other people which may lead to the reduction of COVID-19 infection rate [116]. In addition, Wang et al. showed that remdesivir (a new antiviral drug) and chloroquine can play important roles in the control of COVID-19 infection in vitro [117]. In agreement with this study, Grain et al. conducted a cohort study to find out whether remdesivir is an effective treatment for severe COVID-19 patients or not, and clinical improvements was observed in 36/53 (68%) of patients [118]. According to a study reported by the U.S. National Institute of Allergy and Infectious Diseases (NIAID), remdesivir was associated with a 31% “faster time to recovery.” In this study, about 50% of people who received remdesivir were relieved from the hospital after 11 days vs. 15 days for people who received placebo. The death rate was also reduced among people who received remdesivir (8%) vs. placebo (11%). This result, while encouraging, was not statistically significant [119]. Apart from these investigations, Luo et al. suggested that tocilizumab can be a good choice for treatment of COVID-19 patients with the risk of cytokine storm and also repeated dose of tocilizumab is recommended for patients with escalated IL-6 rate [120]. After all, a reliable and sensitive detection technique combined with effective treatment strategies can alleviate the infection rate of COVID-19.

### 4.5. Gene Mutations Should Be Considered

Beside all the abovementioned findings in molecular detection methods and the type of specimens, gene mutations of the virus should also be considered, as recent reports have indicated a number of mutations in the SARS-CoV-2 genome [121–124]. SARS-CoV-2's spike (S) protein, which consists of two domains called S1 and S2, is the most predominant contributor to the infection of target cells, by using ACE-2 as its main receptor. S1 plays an important role in the receptor binding, while S2 mediates subsequent membrane fusion [125–127]. D614G is one of these recent mutations, which has been shown to be increased over time. Also, this mutation is located in the C-terminal regain of the S1 domain [121, 128]. Zhang et al. analysis demonstrated that although D614G mutation was not observed in February (among 33 sequences), it was increasingly detectable through April (65%) and May (70%). In addition, these scientists also noted that this mutation in SARS-CoV-2's spike protein contributes to the increases in transmissibility of the virus [129]. Moreover, it has been shown that the mentioned mutation can result in escalations of viral loads in SARS-CoV-2-infected individuals [121].

## 5. Conclusions

Among the COVID-19 patients who had confirmed positive PCR results, most of them showed fever or cough as the major clinical signs; diarrhea, headache, and fatigue were less common between COVID-19 patients. Throat, oropharyngeal, and nasopharyngeal swabs were the most common clinical samples using PCR-confirmed COVID-19 patients. Since the SARS-CoV-2 pandemic has caused worldwide concern and the lack of a well-managed global control over the situation has caused many deaths, it is crucial to update the molecular diagnostics guidelines for handling the situation. That is possible by gaining an understanding of the available advances on assays for the detection of SARS-CoV-2 infection. The capability of these assays, including RT-R-PCR, to provide correct results is not at full potential, and the false-negative results may make it harder to control the situation. However, the selection of the most efficient primer-probe for target genes and samples containing enough viral loads to search for the existence of SARS-CoV-2 helps detecting the virus on time. Despite the fact that the spread of the pandemic has been hindered to some extent and the world restriction measurements have been advanced to the vaccination and treatment stage, the sufficiency of the vaccines and treatments is yet to be understood. So, early detection of infected individuals is still the key to the restriction of the pandemic. The results of this study pointed out the importance of the criteria (i.e., using RT-real-time PCR as the detection method, most reliable samples, and the onset symptoms) and that considering them can lead to early detection of the infected patients and management of the crisis.

## Figures and Tables

**Table 1:** The clinical and molecular results of the conducted studies.

Author	Number of patients	Clinical symptoms	Molecular evaluation				Ref
			Sample type	PCR result (%)		Target gene	
				+	−		
Young	18	Fever: 13 (72%) Cough: 15 (83%) Shortness of breath: 2 (11%) Rhinorrhea: 1 (6%) Sore throat: 11 (61%) Diarrhea: 3 (17%)	Whole blood, stool, and urine samples; nasopharyngeal swabs	18 (100%)	—	—	[20]
Liu	12	Fever (10/12) Cough (11/12) Myalgia (4/12) Chills (5/12) Nausea or vomiting (2/12) Diarrhea (2/12)	Bronchoalveolar lavage fluid (BALF) and throat swabs samples were collected from 10 patients Blood and plasma samples	12 (100%)	—	ORF1ab and N genes of 2019-nCoV	[21]
To	12	—	Saliva	11 (91.7%)	1 (0.03%)	S gene	[22]
Cai	10	Fever: 8 (80%) Cough: 6 (60%) Sore throat: 4 (40%) Stuffy nose: 3 (30%) Sneezing and rhinorrhea 2 (20%)	Nasopharyngeal and throat swabs, fecal samples (6 patients), and urine and serum samples (5 patients)	10 (100%)	—	—	[23]
Guan	1099	Fever (43.8% on admission and 88.7% during hospitalization) Cough (67.8%) Diarrhea (3.8%)	Nasal and pharyngeal swab	1099 (100%)	—	—	[24]
Tian	262	Fever (82.1%) Cough (45.8%) Fatigue (26.3%) Dyspnea (6.9%) Headache (6.5%)		262 (100%)	—	—	[25]
Zou	18	Fever	Nasal swabs and throat swabs	18 (100%)	—	N and Orf1b genes of SARS-CoV-2	[26]
Dai	6	COVID-19 positive: Case 1: fever and headache Case 2: fever, cough, and myalgia Case 3: pharyngalgia and fever Case 4: fever/diarrhea twice Other diseases: Case 1: cough, chest pain, and wheezing Case 2: chest pain	COVID-19 Positive: Case 1: respiratory tract Case 2: blood Case 3: pharyngeal swabs Case 4: blood specimens Other diseases: Respiratory tract	4 (66.7%)	2 (33.3%)	—	[27]
Huang	59	Fever: 40/41 (98%), cough: 31/41 (76%), myalgia or fatigue: 18/41 (44%), sputum production: 11/39 (28%), headache: 3/38 (8%), hemoptysis: 2/39 (5%), diarrhea: 1/38 (3%), and dyspnea: 22/40 (55%)	Blood, serum, nasal, and pharyngeal swabs, BALF, sputum, and bronchial aspirates	41 (69%)	18 (31%)	NP gene	[28]
Huang	84	Fever: 32 (94.1%), cough: 17 (50%), myalgia or fatigue: 22 (64.7%), diarrhea: 5 (14.7%), and headache: 2 (5.9%)		34 (40%)	50 (60%)	—	[29]
Li	425	—	Respiratory specimen BALF	425 (100%)	—	Open reading frame 1a or 1b and nucleocapsid protein	[30]
Chang	13	Fever: 12, cough: 6 (46.3%), upper airway congestion: 8 (61.5%), myalgia: 3 (23.1%), and headache: 3 (23.1%)	Throat swab	13 (100%)	—	—	[31]
Wang	138	Fever: 136 (98.6%) Fatigue: 96 (69.6%) Dry cough: 82 (59.4%) Anorexia: 55 (39.9%) Myalgia: 48 (34.8%) Dyspnea: 43 (31.2%) Expectoration: 37 (26.8%) Pharyngalgia: 24 (17.4%) Diarrhea: 14 (10.1%) Nausea: 14 (10.1%) Dizziness: 13 (9.4%) Headache: 9 (6.5%) Vomiting: 5 (3.6%) Abdominal pain: 3 (2.2%)	Throat swab	138 (100%)	—	ORF1ab and nucleocapsid protein (N)	[32]
Yang	149	Fever: 114/149 (76.5%), cough: 87/149 (58.4%), expectoration: 48/149 (32.2%), vomiting: 2/149 (1.3%), dyspnea: 2/149 (1.3%), chest pain: 5 (3.36%), chest tightness: 16 (10.74%), chills: 21 (14.09%) Sore throat: 21 (14.09%) Diarrhea: 11 (7.38%) Muscle pain: 5 (3.36%) Snotty: 5 (3.36%)	Nasal and pharyngeal swab specimens or induced sputum	149 (100%)	—	—	[33]
Xu	50	Fever Cough: 20/50 (40%)/ Expectoration: 7/50 (14%) Sore throat: 4/50 (8%) Headache: 5/50 (10%) Fatigue: 8/50 (16%) Muscle ache: 8/50 (16%) Chest tightness and dyspnea: 4/50 (8%) Gastrointestinal reaction: 1/50 (2%)		50 (100%)	—	—	[34]
Xu	62	Fever: 48/62 (77%), cough: 50/62 (81%), expectoration: 35/62 (56%), headache: 21/62 (34%), myalgia or fatigue: 32/62 (52%), diarrhea: 3/62 (8%), hemoptysis: 2/62 (3%), and shortness of breath: 2/62 (3%)	Sputum and throat swabs	62 (100%)	—	—	[35]
Xie	19	Fever: 14 cases Cough: 13 cases Fatigue: 9 cases Diarrhea: 3 cases	Oropharyngeal swab, blood, urine, and stool	9 (47.4%)	10 (52.6%)	ORF1b and N	[36]
Chen	99	Fever: 82 (83%), cough: 81 (82%), shortness of breath: 31 (31%), muscle ache: 11 (11%), confusion: 9 (9%), headache: 8 (8%), sore throat: 5 (5%), rhinorrhea: 4 (4%), chest pain: 2 (2%), diarrhea: 2 (2%), and nausea and vomiting: 1 (1%)	Throat swab/blood	99 (100%)	—	—	[37]
Shi	81	Fever: 59 (73%) Dyspnea: 34 (42%) Chest tightness: 18 (22%) Cough: 48 (59%) Sputum: 15 (19%) Rhinorrhea: 21 (26%) Anorexia: 1 (1%) Weakness: 7 (9%) Vomiting: 4 (5%) Headache: 5 (6%) Dizziness: 2 (2%) Diarrhea: 3 (4%)	Throat swab specimens	81 (100%)	—	Envelope gene	[38]
Xu	90	Fever: 70 (78%) Cough: 57 (63%) Sputum production: 11 (12%) Fatigue weakness: 19 (21%) Myalgia: 25 (28%) Sore throat: 23 (26%) Chills: 6 (7%) Headache: 4 (4%) Diarrhea: 5 (6%) Nausea: 5 (6%) Vomit: 2 (2%) No obvious symptoms: 6 (7%)		90 (100%)	—	—	[39]
Zhang	178	—	Oral swabs, anal swabs, and blood of these patients, 8 were oral swabs positive (53.3%), 4 were anal swabs positive (26.7%), 6 blood positives (40%), and 3 serum positives (20%). Two patients were positive by both oral swab and anal swab, yet none of the blood positive was also swab positive	In the first investigation: 39 (100%) patients In the second investigation: 139 (100%) patients	—	S gene	[40]
Ai	1014	Fever and dry cough	Throat swab	601 (59%)	413 (41%)	—	[41]
Bergheim	121	Fever: 74 cases (61%) Cough: 58 cases (48%) Sputum production: 20 cases (17%)	Bronchoalveolar lavage, endotracheal aspirate, nasopharyngeal swab, or oropharyngeal swab.	121 (100%)	—	—	[42]
Chen	29	The main symptoms of 2019-nCoV pneumonia was fever (28/29) with or without respiratory and other systemic symptoms		29 (100%)	—	—	[43]
Chung	21	Fever: 14 cases (67%) Fatigue: 3 cases (14%) Headache: 3 cases (14%) Cough: 9 cases (43%) Muscle soreness: 3 cases (14%) Nausea: 1 case (5%) No obvious symptoms: 2 cases (10%)	Bronchoalveolar lavage, endotracheal aspirate, nasopharyngeal swab, or oropharyngeal swab	21 (100%)	—	—	[44]
Fang	51	Fever or acute respiratory symptoms of unknown cause	Throat swab (45 patients) or sputum samples (6 patients)	51 (100%)	—	—	[45]
Kui	137	Fever (112/137, 81.8%), expectoration 6 (4.4%), hemoptysis 7 (5.1%), coughing (66/137, 48.2%), and muscle pain or fatigue (44/137, 32.1%), with other, less typical initial symptoms observed at low frequency, including heart palpitations, diarrhea, and headache	Respiratory tract samples	137 (100%)	—	ORF1ab/N	[46]
Li	53 cases	In 51 cases: Fever: 46 (90.2%) Fatigue and poor appetite: 3 (5.9%) Cough: 1 (2.0%) No symptoms: 1 (2.0%)	Oropharyngeal swab	51 (96.3%)	2 (3.7%)	—	[47]
Li	54	Fever, chills, cough, fatigue, and chest distress. Some other symptoms were headache, myalgia, dyspnea, diarrhea, nausea, and vomiting	Pharyngeal swab	31 (57%)	23 (43%)	—	[48]
Liu	30	Fever in 23 patients (76.67%), headache in 16 patients (53.33%), fatigue or myalgia in 21 patients (70%), nausea, vomiting, or diarrhea in 9 patients (30%), cough in 25 patients (83.33%), and dyspnea in 14 patients (46.67%)		30 (100%)	—	—	[49]
Liu	78	Cough, fever, and respiratory tract infection symptoms	Nasopharyngeal swabs	78 (100%)	—	—	[50]
Song	51	The most common symptoms were fever (49 of 51, 96%) and cough (24 of 51, 47%). Other symptoms included myalgia or fatigue (16 of 51, 31%), mild headache and dizziness (eight of 51, 16%), and diarrhea (five of 51, 10%)		51 (100%)	—	—	[51]
Wu	80	61/80 (76%) of patients had fever, 58/80 (73%) of patients had cough, 11/80 (14%) of patients had expectoration, 5/80 (6%) of patients had chest pain, 13/80 (16%) of patients had muscle ache, 7/80 (9%) of patients had abdominal pain or diarrhea, 9/80 (11%) of patients had pharyngeal discomfort, 8/80 (10%) of patients had dizziness or headache, 7/80 (9%) of patients had dyspnea, and 3/80 of patients (4%) had blood in sputum	Throat swabs or lower respiratory tract blood samples	80 (100%)	—	—	[52]
Xia	30	—	Respiratory or blood specimens Tears and conjunctival secretions (conjunctival swab) Sputum sample	1 patient had positive results in conjunctival swab samples and sputum samples (3%)	29 patients had negative conjunctival swab samples (97%)	—	[53]
Xie	167	Case 1: fever and mild cough Case 2: fever of 39°C Case 3: cough, dizziness, and debility, but had no fever Case 4: fever Case 5: fever		In 7 (4%), CT was initially negative while RT-PCR was positive/in 155 (93%), both RT-PCR and CT were concordant for COVID-19	5 (3%) initially had negative RT-PCR but positive chest CT/in 155 (93%), both RT-PCR and CT were concordant for COVID-19	—	[54]
Zhang	140	The most commonly experienced symptoms were fever (91.7%), followed by cough (75%), fatigue (75%), and chest tightness or dyspnea (36.7%). 39.6% of them complained of gastrointestinal symptoms, including nausea, diarrhea, poor appetite, abdominal pain, belching, and emesis	Pharyngeal swab	140 (100%)	—	—	[55]
Zhao	101	Fever: 79 (78.2%) Cough: 63 (62.4%) Myalgia or fatigue: 17 (16.8%) Sore throat: 12 (11.9%) Dyspnea: 1 (1.0%) Diarrhea: 3 (3.0%) Nausea and vomiting: 2 (2.0%) More than one symptom: 67 (66.3%) None: 2 (2.0%)		101 (100%)	—	—	[56]
Feng	15	Five of the 15 children were febrile, and 10 were asymptomatic	Nasal or pharyngeal swab samples	15 (100%)	—	—	[57]
Li	83	Fever 72 (86.7%), cough 65 (78.3%), expectoration 15 (18.1%), and myalgia 15 (18.1%). Less common symptoms were headache, dyspnea, abdominal pain/diarrhea, pharyngeal discomfort, and chest pain		83 (100%)	—	—	[58]
Kim	28	Cough (28.6%), sore throat (28.6%), fever (25.0%), diarrhea (10.7%) myalgia, and headache (7, 25.0%)	RT-PCR: nasopharyngeal and oropharyngeal swab with or without sputum	28 (100%)	—	E gene	[59]
Chen	42	Fever (85.71%), dry cough (52.38%), fatigue (52.38%), myalgia (23.81%), and dyspnea (21.43%). Sputum production (16.67%), pharyngalgia (14.29%), headache or dizziness (11.9%), diarrhea (16.67%), abdominal pain (11.95%), nausea (9.52%), and vomiting (7.14%)	RT-PCR: pharyngeal swab, stool, and urine	Pharyngeal swab 42 (100%)	—	—	[60]
				Stool 28 (66.67%)	14 (33.33%)		
				Urine 32 (76.19%)	10 (23.81%)		
Huang	30	Fever (84%), cough (72%), chills (24%), fatigue (40%), myalgia (12%), and loss of appetite (40%)	RT-PCR	30 (100%)	—	—	[61]
Cheng	33	Fever (72.7%), cough (63.6%), muscle ache (27.3%), diarrhea (9.1%), sore throat (9.1%), and sputum (27.3%)	Real-time RT-PCR: throat swab	11 (33.3%)	22 (66.7%)	—	[62]
Himoto	21	—	Real-time RT-PCR	6 (29%)	15 (71%)	—	[63]
Lei	14	Fever (50%), cough (50%), headache (14%), fatigue (21%), body soreness (7%), and diarrhea (21%)	Real-time RT-PCR: sputum	14 (100%)	—	—	[64]
Caruso	158	Fever (61%), cough (56%), and dyspnea (33%)	Real-time RT-PCR: nasopharyngeal and oropharyngeal swabs	62 (39%)	96 (61%)	—	[65]
Chan	15	—	Real-time RT-PCR respiratory tract specimen, urine, rectal swab, and feces	15 (100%)	—	RdRp, Hel, S, and N genes of SARS-CoV-2	[66]
Chen	249	Fever (87.1%), cough (36.5%), fatigue (39 (15.7%)), dizziness and headache (11.2%), sore throat (6.4%), rhinorrhea (6.8%), diarrhea (3.2%), and inappetence 8 (3.2%)	PCR: upper respiratory tract specimens throat swab samples	249 (100%)	—	—	[67]
Albano	65	—	RT-PCR	5 (7.7%)	—	—	[68]
Chao Yan	130	—	Bronchoalveolar swabs and fluid	58 (44.6%)	72 (55.4%)	orf1ab-4 and S-123	[69]
Liang Su	14	Fever (57.1%), cough (35.7%), chest tightness/pain (21.4%), fatigue (21.4%), and sore throat (7.1%)	Sputum and nasopharyngeal swabs	14 (100%)	—	ORF1ab/N	[70]
Yun Ling	66	Fever, cough, and dyspnea	Oropharyngeal swabs stool, urine, and blood specimens	11 (17%)	55 (83%)	—	[71]
Kelvin Kai-Wang To	30	Fever in 22 patients (96%), followed by cough in five (22%), chills in four (17%), and dyspnea in four (17%)	Blood, urine, posterior oropharyngeal saliva, and rectal swabs	23 (77%)	7 (23%)	In-house reverse transcriptase quantitative PCR (RT-qPCR) targeting the SARS-CoV-2 RNA-dependent-RNA-polymerase-helicase gene region	[72]
Lu Lin	95	Fever or respiratory symptoms Diarrhea: 23 (24.2%)/anorexia: 17 (17.9%)/nausea: 17 (17.9%)/vomiting: 4 (4.2%)/vomiting: 2 (2.1%)	Pharyngeal swab	58 (61%)	37 (39%)	ORF1ab/N	[73]
Yinxiaohe Sun	788	—	Sputum, nasopharyngeal swabs, or throat swabs	54 (7%)	734 (93%)	—	[74]
Chunqin Long	36	Fever (36/36, 100%), cough (27/36, 75.0%), myalgia or fatigue (14/36, 38.9%), and nausea or diarrhea (6/36, 16.6%)		30 (83%)	6 (17%)	—	[75]
Feng Pan	21	Fever (86%) and cough (57%)		21 (100%)	—	—	[76]
Rui Liu	4880	Fever (100%), cough and hard breath, or both	Nasal and pharyngeal swabs	1875 (38.5%)	3005 (61.5%)	1ab (ORF1ab) and nucleocapsid protein (NP) genes	[77]
Xiaobing Wang	1012	Fever (761 of 1012, 75.2%), cough (531 of 1012, 52.4%), dyspnea (231 of 1012, 22.8%), expectoration (220 of 1012, 21.7%), chills (182 of 1012, 18.0%), myalgia (170 of 1012, 16.8%), headache (152 of 1012, 15.0%), diarrhea (152 of 1012, 15.0%), sore throat (144 of 1012, 14.2%), nasal congestion (69 of 1012, 6.9%), runny nose (57 of 1012, 5.6%), abdominal pain (37 of 1012, 3.7%), and vomiting (36 of 1012, 3.6%)	Sputum or nasopharynx swabs	311 (30.5%)	701 (69.5%)	—	[78]
Jian Wu	80	Fever: 63 (78.75%), cough: 51 (63.75%), and shortness of breath: 30 (37.5%). Muscle ache: 18 (22.50%), headache and mental disorder symptoms: 13 (16.25%), diarrhea: 1 (1.25%), and chest pain: 3 (3.75%)	Nose swab and/or throat swab	80 (100%)	—	Nucleocapsid (N) gene and open reading frame lab (ORF1ab) gene	[79]

## Data Availability

The data are available from Jonathan R. Dimmock on reasonable request.
